# Profiling skin microbiota in an underrepresented population: Indonesian children with atopic dermatitis and controls

**DOI:** 10.3389/fmed.2026.1697420

**Published:** 2026-02-12

**Authors:** Raden Mohamad Rendy Ariezal Effendi, Willemijn C. A. M. Witkam, Reiva Farah Dwiyana, Oki Suwarsa, Robert Kraaij, Chrysanti Murad, Hok Bing Thio, Tamar Nijsten, Luba M. Pardo

**Affiliations:** 1 Department of Dermatology, Erasmus MC University Medical Center Rotterdam, Rotterdam, Netherlands; 2 Department of Dermatology, Venereology, and Aesthetics, Faculty of Medicine, Universitas Padjadjaran - Dr. Hasan Sadikin General Hospital, Bandung, Indonesia; 3 Department of Internal Medicine, Erasmus MC, University Medical Center Rotterdam, Rotterdam, Netherlands; 4 Division of Microbiology, Department of Biomedical Sciences, Faculty of Medicine, Universitas Padjadjaran, Bandung, Indonesia

**Keywords:** amplicon sequence variant, atopic dermatitis, children, Indonesia, skin microbiota, underrepresented

## Abstract

**Introduction:**

The skin microbiome plays a central role in the pathogenesis of atopic dermatitis (AD), but most studies have focused on high-income populations of European ancestry. Microbiome data from tropical and developing regions remains limited. In Asia, microbiome research has similarly centered around developed countries, leaving populous developing countries like Indonesia underrepresented. The aim was to profile the cutaneous bacterial microbiota of children with AD from Indonesia and compare it with controls.

**Methods:**

Skin swabs were collected from lesional sites of 111 children aged 4–18 years with AD and from the forearms of 107 controls, all attending Pediatric Dermatology Clinic of Dr. Hasan Sadikin General Hospital, an urban tertiary-care referral center in Bandung, West Java, Indonesia. AD was diagnosed using Hanifin–Rajka criteria, while controls had non-atopic, non-inflammatory dermatological conditions. Cutaneous bacterial microbiota was profiled using 16S rRNA sequencing with amplicon sequence variant (ASV) level analysis using DADA2 pipeline. Data was analyzed after quality control to estimate alpha and beta diversities, the later using, permutational multivariate analysis of variance (PERMANOVA) to assess contribution of individual variables to the variation in microbiota composition. Univariable differential abundance was done to analysis the composition of specific bacteria in cases versus controls. Analysis of core microbiota compositions and phylogenetic relationships were explored to identify key taxa associated with AD.

**Results:**

Most children came from families with higher household incomes, and children with AD were younger than controls (mean age 8.35 ± 3.51 vs. 9.91 ± 3.79 years, *P* = 0.002). Lesional AD skin showed a significantly reduced alpha diversity and a marked overrepresentation of *Staphylococcus aureus* and *Staphylococcus epidermidis*. Less commonly reported genera, including *Acetobacter* and *Gluconobacter*, were enriched in cases, potentially reflecting environmental exposure in this cohort. PERMANOVA revealed that case–control status, family income, maternal atopy, maternal education and DNA concentration significantly influenced microbial composition. Phylogenetic analysis showed a clear lineage-level distinction between *Staphylococcus* ASVs.

**Conclusion:**

Our findings reveal distinct microbial profiles in children with AD from a tropical, underrepresented population with predominantly higher household incomes, and underscore the role of environmental and sociodemographic factors associated with skin microbiota. While generalizability to lower-income or rural populations may be limited, the value of ASV-level analysis lies in its ability to capture both known and less characterized microbial signals.

## 1 Introduction

Atopic dermatitis (AD) is a chronic, relapsing inflammatory skin disease with a global prevalence range of 4%–20% in children and 2%–10% in adults. Its prevalence varies widely by age, geographic region, and income setting (1–3). The pathogenesis of AD is driven by a complex interplay of genetic, environmental, and immunological factors. Recent insights underscore the pivotal role of the skin bacterial microbiota in its development and exacerbation (1, 4). Understanding the relationship between skin bacterial microbiota and AD progression is essential for advancing treatment approaches and improving our understanding in disease mechanisms, particularly as microbial dysbiosis has been observed to correlate with disease flares (5–8).

Most studies on the skin bacterial microbiota have been conducted in high-income populations of European ancestry. In these populations, the role of *Staphylococcus*, particularly *Staphylococcus aureus* is frequently emphasized as a key contributor to skin dysbiosis in AD (7, 9, 10). This Eurocentric focus restricts our understanding, as microbial diversity and composition can vary significantly across ethnic, geographic populations, socioeconomic settings, life style habits, different climate and environment (3). Epidemiological studies of pediatric AD in Indonesia are limited, but data from a multicenter study from the “Indonesian Pediatric Dermatology Study Group” indicates that AD accounts for about 24% of pediatric skin disease diagnoses in participating centers. This makes AD, the most common dermatosis in Indonesian children (11). As one of the most populous and non-high-income countries in Asia, Indonesia offers a unique epidemiological landscape for AD research, with potentially distinct skin microbial profiles from those of other Southeast Asian populations. Moreover, most individuals have Fitzpatrick skin types III–V [(12, 13) (medium to dark brown skin, with types IV–V most common)], which is different from European studies (type I, II; very white and white) and populations exhibit a wide range of hygiene practices, environmental exposures, and cultural practices that may influence skin microbial composition. This could deepen our understanding on the role of the bacterial microbiota in AD and aid to more generalizable results to diverse global populations, including those in underrepresented, non–high-income regions. To address these gaps, we profiled the bacterial skin microbiota using 16S rRNA sequencing in AD cases and controls in a pediatric Indonesian population (ages 4–18).

## 2 Materials and methods

### 2.1 Study population and design

This cross-sectional study received ethical approval from the Health Research Ethics Committee of Dr. Hasan Sadikin General Hospital, Bandung, West Java, Indonesia. Bandung is the capital city of West Java on the island of Java, the most populous island in Indonesia. It is a highly urbanized city of about 2.5 million inhabitants, with densely populated neighborhoods and marked socio-economic variation. Bandung is located at an elevation of roughly 700 m above sea level and has a tropical climate with a distinct rainy season and relatively cooler temperatures than many lowland Indonesian cities (14).

The study was conducted in compliance with the ethical principles outlined in the World Medical Association Declaration of Helsinki (reference:LB.02.01/X.6.5/111/2022). Written informed consent was obtained from the parents or legal guardians of all participants prior to inclusion.

The study included Indonesian children aged 4–18 years, divided into two groups: AD cases and controls. All participants were recruited from the Pediatric Dermatology Clinic at Dr. Hasan Sadikin General Hospital to ensure environmental and demographic comparability. AD diagnoses were confirmed by Pediatric Dermatologists (R.M.R.A.E and R.F.D) according to Hanifin and Rajka Criteria (HRC) (15) including full body skin examination. Disease severity was assessed using the Scoring Atopic Dermatitis (SCORAD) Index, which categorized participants into three severity groups: mild AD (SCORAD < 25), moderate AD (SCORAD 25–50), and severe AD (SCORAD > 50) (16–18). Eligibility for the AD group required no topical corticosteroid or antibiotic use in the prior 7 days, and no systemic antibiotics, corticosteroids, or probiotics within the past 2 months. Those who did not meet these criteria were excluded. Participants were instructed to avoid using emollients for 24 h and from showering for at least 6 h before sampling.

The control group consisted of children with non-atopic and non-inflammatory dermatological conditions. Controls were recruited from siblings or relatives accompanying children with AD to the pediatric dermatology clinic; children living in the same neighborhoods as AD cases or in other urban neighborhoods within Bandung who were invited for the study through local outreach (e.g., posters, flyers),and non-atopic children attending the pediatric dermatology clinic for other reasons. We excluded participants with any history of atopic disease (e.g., AD, asthma, allergic rhinitis, food allergy), as well as inflammatory or infectious skin disorders.

Each child underwent a structured interview and clinical examination to confirm eligibility. Only those without active skin lesions and not meeting the HRC were included. Inclusion as a control was confirmed by two pediatric dermatologists (R.M.R.A.E and R.F.D) following comprehensive skin examinations. Controls were selected to reflect similar age and sex distributions. Where possible, non-atopic siblings of AD cases were included as non-matched controls to ensure comparable socio-environmental exposures relevant to the skin microbiota. In total, 20 sibling-controls were included in the control group.

Fitzpatrick skin type was assessed clinically for all participants using the standard six-point scale, which ranged from type I (very fair skin that always burns and never tans) to type VI (deeply pigmented skin that never burns; higher types indicated darker skin pigmentation) (12, 19). Comprehensive demographic data were collected through structured interviews with parents or guardians. Family income was obtained by self-report and grouped using Bandung-adjusted thresholds from the Indonesian Central Bureau of Statistics (Badan Pusat Statistik, BPS), which are based on monthly household income, household size, and typical expenditure, to approximate the local urban socioeconomic distribution (20).

### 2.2 Microbial sample collection

Skin swabs were taken after clinical assessment over a 9-month period (April to December 2022) during regular daytime clinic hours. The skin swabs were collected from both lesional and non-lesional sites for AD participants. Lesional samples were taken from the antecubital or popliteal folds. If no active lesions were present in these areas, samples were taken from the most prominent affected site. Non-lesional samples in AD and controls were collected from the dorsal area of the distal forearm.

Skin samples were collected using flocked swabs with prefilled preservative tube kits (Copan ^®^ 608CS01R; FLOQSwab ^®^ with eNAT ^®^ 1.0 mL). To enhance microbial recovery from this low-biomass surface, swabs were pre-moistened with sterile 0.9% NaCl prior to sampling (21). Each 5 × 5 cm skin area was swabbed for 60 s with firm pressure, first using the moistened side of the swab horizontally across the surface, followed by the dry side vertically.

After sampling, the swab tip was broken into the collection tube, the eNAT ^®^ preservative was released by pressing the internal plunger, and the tube was sealed and shaken to mix. eNAT ^®^ is a guanidine-thiocyanate stabilization medium that lyses microorganisms, inactivates nucleases and pathogens, and preserves nucleic-acid integrity and microbiome composition for up to 30 days at ambient temperature (22). All samples were stored at −80 °C until DNA extraction at the Biomedical Sciences Lab, Faculty of Medicine, Universitas Padjadjaran, Bandung, Indonesia.

Negative controls included 15 air swabs and 4 kit controls from the NextGen platform. Air swabs were collected biweekly by opening the tube and exposing the swab to ambient air for 60 s using the same swab kits as those used for participants.

### 2.3 DNA extraction and 16S rRNA gene polymerase chain reaction amplification and sequencing

DNA was extracted using the QIAamp DNA Microbiome Kit (Qiagen), following the manufacturer’s protocol. Samples were first incubated with Buffer AHL for 30 min, followed by centrifugation and treatment with Buffer RDD and Benzonase at 37 °C for 30 min. After adding Proteinase K and incubating at 56 °C, samples underwent mechanical lysis in Pathogen Lysis Tubes using a FastPrep-24 instrument. The lysate was then combined with Proteinase K, incubated, and mixed with Buffer APL2 and ethanol. This mixture was applied to a QIAamp spin column, washed with Buffers AW1 and AW2, and finally eluted with Buffer AVE to isolate purified DNA and was stored at −80 °C until shipment. In accordance with the material transfer agreement (HK.01.07/H/2918/2023), extracted DNA samples were transported on dry ice to Rotterdam, Netherlands via World Courier*™*, equipped with temperature-monitoring devices to ensure their safety and quality throughout shipment. The V1–V3 variable regions of the 16S rRNA gene were amplified and sequenced on the Illumina NextSeq2000 platform at the Genomics Core Facility of Erasmus University Medical Center, Rotterdam, the Netherlands (Erasmus MC). Positive controls were processed alongside study samples to monitor library preparation, sequencing, and taxonomic assignment, including a commercial microbial reference standard and an internal reference standard provided by the Genomics Core Facility in the Netherlands (internal communication).

### 2.4 Analysis

#### 2.4.1 Bioinformatic analysis

Raw reads from the Illumina NextSeq2000 platform were demultiplexed using custom scripts in QIIME1 (version 1.9.1) to generate sample-specific FASTQ files (23). Primer sequences were trimmed with TagCleaner (v0.16) (24). The DADA2 pipeline (v1.16) in R (version 4.0.0) was applied to process and filter the reads, allowing for exact amplicon sequence variant (ASV) inference, which minimizes errors without clustering at a similarity threshold (25). After denoising, chimeric sequences were removed using DADA2, and the resulting chimera-free ASV dataset was used for all downstream analyses. ASVs represent unique biological sequences inferred directly from reads, offering higher resolution and reproducibility compared to traditional operational taxonomic units (OTUs) (25, 26). Taxonomic classification was performed with the RDP naïve Bayesian classifier within DADA2 (27), using the SILVA SSU rRNA reference database (release 138.1) (28). The ASV read counts and accompanying taxonomic information were compiled into a phyloseq object to generate abundance and taxonomy tables (29). Primary analyses were performed at the ASV level. For descriptive community summaries, the ASV was aggregated to the phylum and genus level and displayed as relative-abundance bar plots.

#### 2.4.2 Cutaneous bacterial microbiota data filtering

Quality control of the microbiota was done with the prevalence function of the “Decontam” R package (30). This function compares the prevalence of ASVs in true samples and negative controls to estimate the probability of an ASV to be a contaminant. According to the evaluation histogram (Supplementary Figure 1), we applied a threshold of *P* = 0.45 to identify and exclude potential contaminants. A minimum read depth threshold was selected based on the plateau of the rarefaction curve and was set at 2,000 reads (Supplementary Figure 2). ASVs were further filtered using a 20% prevalence threshold. The filtering steps are summarized in the flow diagram presented in Supplementary Figure 3.

#### 2.4.3 Data analysis

Demographic characteristics of study participants were presented as proportions and compared between cases and controls using Chi-squared tests for categorical data and both independent *t*-tests and Mann-Whitney U tests for normally and non-normally distributed continuous data.

To compare bacterial profiles between AD cases and controls, we estimated alpha diversity for each sample using Chao1 and Shannon indexes. The Chao1 index estimates sample richness by calculating the number of ASVs in each group, while the Shannon index reflects both richness and evenness by considering the relative distribution of sequences among ASVs (31). Group differences in alpha diversity were evaluated with the Kruskal-Wallis test, and *p*-values were adjusted for pairwise comparisons. Univariable and multivariable linear regression models were used to evaluate associations between AD status and alpha diversity. The multivariable models were adjusted for age, body mass index (BMI) group, gestational age (full-term or preterm), parental education (categorized as intermediate for secondary school and vocational training, and high for university degrees or higher), family income (high or very high), birth method (cesarean section or vaginal delivery), breastfeeding status (exclusive or non-exclusive), feeding habits (hand-fed or spoon-fed), parental history of atopy (yes or no), and DNA concentration. Multiple testing correction was applied using the Benjamini-Hochberg method.

Beta diversity was calculated using Bray-Curtis distance (32) and plotted using Principal Coordinate Analysis (PCoA). The distances were calculated after zero imputation and standardization using Hellinger standardization For the zero imputation we used the multiplicative method to avoid effects of sparsity and compositional bias (33, 34). Subsequently, PERMANOVAwas used to evaluate the association between individual variables and its contribution to the overall variation of the microbiota composition (function “adonis2,” R package “vegan,” version 2.6.4 with Bray Curtis distances) (35). The variables tested included skin type, age, BMI, gestational age, parental education, family income, parental history of atopy, birth method, breastfeeding status, feeding habits, and DNA concentration. Variables that showed a statistically significant association in univariable models were included in the final multivariable PERMANOVA, using the same set of variables as in the alpha diversity models.

To assess differences in the bacterial microbiota composition between cases and controls, we first evaluated differences in relative abundances visually at genus levels using bar plots. Then, we compared the core microbiota (ASVs present in at least 75% of the cases or controls) at ASV level using heatmaps. To identify which taxa were differentially abundant in cases compared to controls (reference) while adjusting for potential confounders and bias, we employed Analysis of Compositions of Microbiomes with Bias Correction (ANCOMBC2, package “ancombc” version 2.4.0), which applies a bias correction approach to account for compositional data issues and provides adjusted estimates for differential abundance (36). The differential abundances were measured in log-fold change (LFC) values. The model included the same set of variables, including age, BMI group, parental education, family income, gestational age, birth method, exclusive breastfeeding, feeding habit, parental history of atopy, and DNA concentration. Multiple testing correction was performed using the Benjamini–Hochberg method, and statistical significance was defined as an adjusted *p*-value < 0.05. To minimize noise from rare taxa, we performed the ANCOMBC2 analysis with a prevalence threshold of 20% at both genus and ASV levels.

To investigate the phylogenetic relationships of the relevant bacteria that could not be unequivocally be assigned to species level by the DADA2 pipeline, a targeted phylogenetic analysis was conducted. We focused on ASVs belonging to the genus *Staphylococcus*. We used the read sequences of ASVs that were relevant (core microbiota analysis; ANCOMBC) and compared them with uploaded references in the National Center for Biotechnology Information (NCBI) nucleotide database, using the Basic Local Alignment Search Tool (BLASTn), with the “megablast” function (37). The top BLAST hits were selected based on percentage identity. The species names were added to the taxonomy names of the ASVs with 100% identity to a reference species sequence. In addition, we constructed a phylogenetic tree, using the Neighbor-Joining method in MEGA version 11 (38). Using sequences of several *Staphylococcus* species downloaded from GenBank, similar to a previous publication (39). ASVs that we called to a species level by our phylogenetic analysis have the word likely in the results. All analyses were conducted using R software, version 4.3.2 (40). All plots and statistical analyses were generated using the packages “phyloseq” version 1.42.0 and “microbiome” version 1.24.0.

#### 2.4.4 Sensitivity analyses in AD cases

We first conducted an exploratory comparison of lesional and non-lesional skin samples from the same AD patients. This analysis aimed to complement the main case–control comparisons by assessing site-specific microbial differences within individuals.

Second, we compared the alpha diversity in AD cases separated by AD severity. For this we used the SCORAD scores stratified as mild, moderate and severe (mild < 25, moderate 25–50, severe > 50) using covariate-adjusted multivariable linear models, and using the mild group as reference.

Third, we also looked into toilet seat dermatitis (TSD). TSD is considered a context-specific phenotype of AD in this population (41) potentially influenced by bidet use, exposure to chemical cleansing products, prolonged toilet-seat contact, and environmental factors such as high humidity. A subset of AD participants presenting with clinical signs of toilet seat dermatitis (TSD) was compared to AD cases without TSD to assess whether a distinct microbial pattern is associated with TSD. The groups were evaluated for compositional differences (visual differences in relative abundances) and the multivariable adjusted alpha diversities. In cases of TSD, swabs were not taken from the buttock unless it was the most prominent lesional site, as defined by the sampling approach used in this study.

## 3 Results

Skin swabs were collected from 218 participants, namely: 111 AD cases and 107 controls. A total of 222 swabs were collected from the AD cases – 111 from lesional sites and 111 from non-lesional sites, In the main analysis, lesional skin samples from AD cases were compared with non-lesional skin samples from controls. Including the swabs from the controls, negative controls, and kit controls, this resulted in a total of 348 samples with 4871 ASVs. The AD group was almost 2 years younger than the control group (mean age of 8.35 ± 3.51 vs. 9.91 ± 3.79 years, *P* = 0.002, Table 1). The distribution of skin colors was similar between cases and controls, with most participants having Fitzpatrick skin type IV (55.9% and 43.9%, respectively).

**TABLE 1:** Participants with skin swabs.

Demographics	Lesional AD (*n* = 111)	Controls (*n* = 107)	*P*-value
Age (years) mean ± SD	8.35 ± 3.51	9.91 ± 3.79	0.002*
Age (years) median (IQR)	8.00 (6.0)	10 (6.0)	0.003*
Biological sex			0.95
Male	52 (46.8%)	52 (48.6%)	
Female	59 (53.2%)	55 (51.4%)	
Skin type			0.35
III	19 (17.1%)	21 (19.6%)	
IV	62 (55.9%)	47 (43.9%)	
V	30 (27.0%)	39 (36.4%)	
BMI			0.07
Normal	77 (69.4%)	87 (81.3%)	
Overweight	34 (30.6%)	20 (18.7%)	

Statistical significance for differences between groups is marked with * for *p* < 0.05.

Based on the bacterial profiles of the negative controls, *Acetobacter* and *Gluconobacter* were detected in multiple air swab samples, with *Acetobacter* exceeding the 2,000-read inclusion threshold used in our analysis pipeline. In contrast, both genera were detected only once and at low levels in reagent blanks derived from the NextSeq platform kit. *Gluconobacter* appeared only sporadically in a few air swabs and was absent from all reagent-based controls (Supplementary materials).

### 3.1 Bacterial community profiles

**FIGURE 1:**
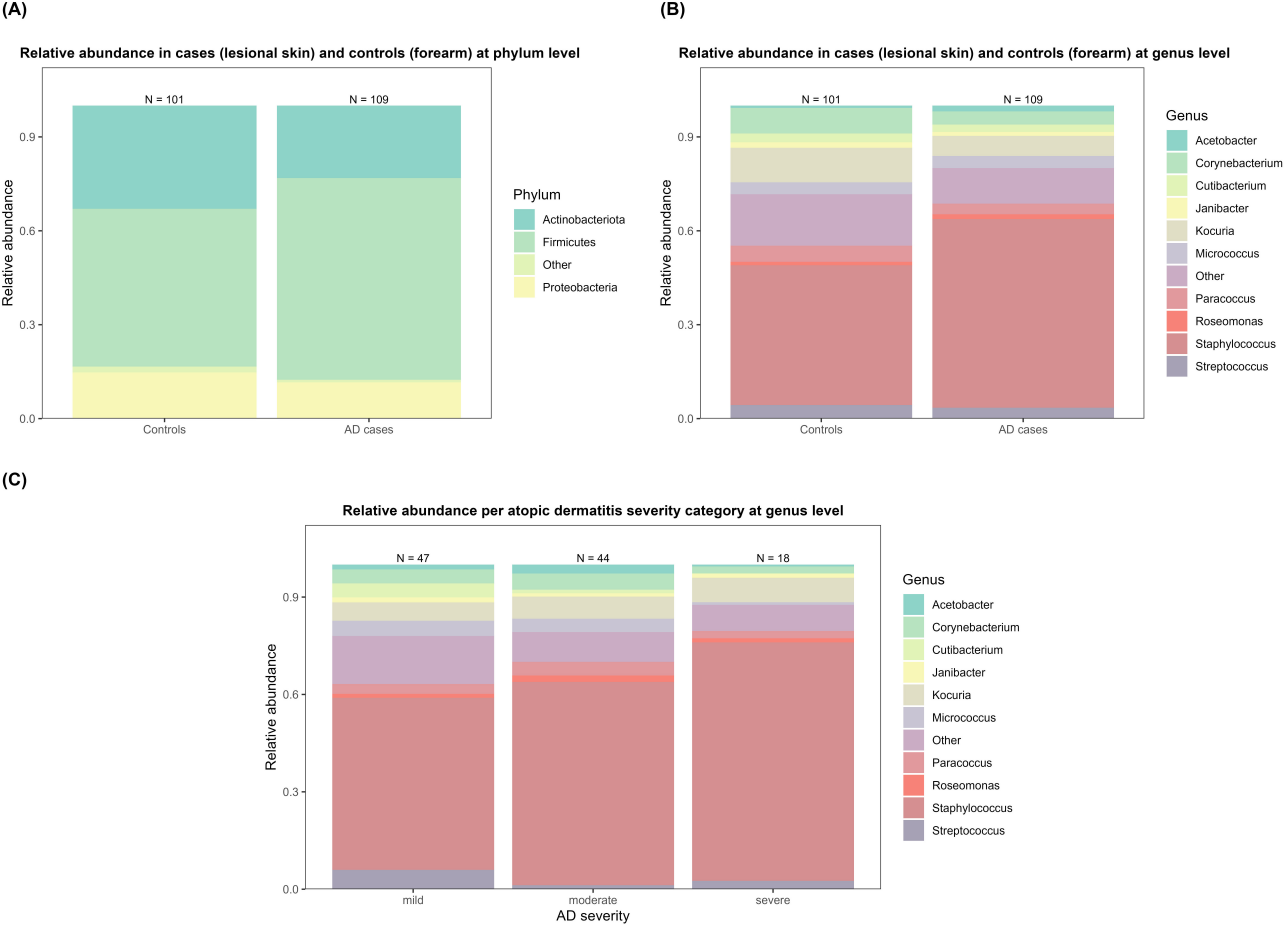
Relative abundance of skin microbiota by atopic dermatitis (AD) status and severity. **(A)** Shows the phylum-level comparison between lesional AD and control samples, with Firmicutes more dominant in AD cases. **(B)** Presents genus-level composition, where *Staphylococcus* was the most abundant genus in both groups, with a higher relative abundance in lesional AD skin. **(C)** Shows genus-level composition stratified by AD severity, demonstrating an increasing abundance of *Staphylococcus* with greater disease severity.

After quality control and filtering, the data were reduced from 4871 ASVs in 348 samples to 211 ASVs across 311 samples, which were included in the final analyses (lesional AD = 109; controls: 101). The microbial composition differed between lesional AD and control samples at both phylum and genus levels (Figure 1). At the phylum level (Figure 1A), *Firmicutes* was slightly more relatively abundant in AD cases compared to controls. Among the genera, *Staphylococcus* was the most dominant in both groups, but its relative abundance was higher in AD cases compared to controls (Figure 1B). Notably, an increase in the relative abundance of *Staphylococcus* was observed with every categorical increase in AD severity (Figure 1C). Differences in microbial composition across various swab sites are shown in Supplementary Figure 4. Stacked bar plots visualizing the relative abundance of *Staphylococcus* by sample type, swab sites, and AD severity are shown in Supplementary Figure 5.

Several ASVs from the *Staphylococcus* genus contributed the most to the core microbiota in both cases and controls (Supplementary Table 1 and Supplementary Figure 6). Notably, ASV1 (likely *S. aureus*) was the most prevalent bacteria in cases and was not present in controls. ASV2 (likely *S. hominis*), ASV4 (likely *S. hominis*), and ASV8 (likely *S. edaphicus)* were observed at a higher prevalence in controls. Overall, there were more bacteria in the control group than in AD cases.

### 3.2 Microbiota diversity

**FIGURE 2:**
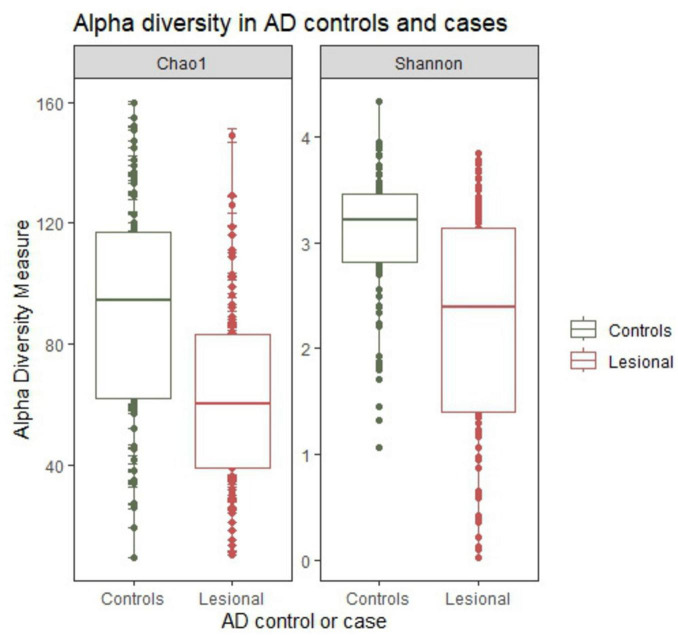
Boxplot showing univariate comparisons of alpha diversity (Chao1 and Shannon index) between controls and atopic dermatitis (AD) lesional skin samples. AD cases exhibited significantly lower alpha diversity in both index (Chao1 and Shannon) compared to controls. Statistical comparisons were performed using linear regression and are reported on the main text.

Alpha diversity was significantly lower in AD cases compared to controls. In the univariable models (Figure 2), both the Chao1(β = −28.61, SE = 4.45, adjusted *p* < 0.001) and Shannon (β = −0.71, SE = 0.15, adjusted *p* < 0.001) showed reduced diversity in cases. AD remained significantly negatively associated with both alpha diversity indexes in the multivariable analyses (Chao1:β = −19.18, SE = 4.37, adjusted *p* < 0.001 and Shannon:β = −0.71, SE = 0.15, adjusted *p* < 0.001, Table 2). Additionally, increasing age was independently associated with lower alpha diversity in both models (Chao1: β = −1.68, SE = 0.49, adjusted *p* = 0.002; Shannon: β = −0.06, SE = 0.02, adjusted *p* < 0.001).

**TABLE 2:** Multivariable linear regression of factors associated with alpha diversity.

Tested exposure	Chao1 richness			Shannon diversity		
	β	SE	Adjusted *p*	β	SE	Adjusted *p*
Lesional cases (ref: non-lesional)	−19.18	4.37	<0.001***	−0.71	0.15	<0.001***
Age in years	−1.68	0.49	0.002*	−0.06	0.02	0.001***
Father education high (ref: intermediate)	−2.0	5.82	0.792	−0.01	0.2	0.967
Mother education high (ref: intermediate)	−3.82	5.63	0.647	−0.11	0.19	0.663
Family income very high (ref: high)	−11.64	4.08	0.01**	−0.31	0.14	0.069
Birth method section caesarea (ref: vaginal)	−3.21	3.94	0.647	−0.15	0.13	0.572
Exclusive breastfeed (ref: no)	−0.83	3.42	0.808	0.07	0.12	0.663
Feeding habit – hand feed (ref: spoon feed)	−2.75	3.76	0.647	−0.08	0.13	0.663
Atopy mother yes (ref: no)	−6.44	4.05	0.212	−0.07	0.14	0.663
Atopy father yes (ref: no)	1.58	4.18	0.792	0.09	0.14	0.663
DNA concentration	−0.87	0.2	<0.001***	−0.03	0.01	<0.001***

Multivariable linear regression models assessing associations between tested exposures and alpha diversity metrics (Chao1 richness and Shannon diversity). Beta coefficients (β), standard errors (SE), and adjusted *p*-values are reported. Models were adjusted for relevant demographic, clinical, and technical covariates. Statistically significant associations (adjusted *p*-value) are marked with * for *p* < 0.05, ** for *p* < 0.01, and *** for *p* < 0.001.

In Supplementary Figure 7, the PCoA plot showed substantial overlap between AD cases and controls, with AD samples slightly more dispersed). In the unadjusted PERMANOVA analyses (one variable at a time), we found significant contributions of AD case status (R^2^ = 2.30%, *p* = 0.001), maternal education (R^2^ = 1.23%, *p* = 0.039), family income (R^2^ = 3.05%, *p* = 0.002), maternal atopy (R^2^ = 2.66%, *p* = 0.008), and DNA concentration (R^2^ = 4.98%, *p* < 0.001) to the overall variance of the microbiota composition. In the adjusted analyses, “AD case status” was the variable that contributed the most to the variation of the skin microbiota, although this was lower than in the univariable analysis. In addition, age, mother education, family income, atopy of the mother and DNA concentration were also significant (Table 3).

**TABLE 3:** Unadjusted and adjusted permutational multivariate analysis of variance (PERMANOVA) results for microbiota composition.

Tested main exposure	Unadjusted R^2^ (%)	Unadjusted (*p*-value)	Adjusted R^2^ (%)	Adjusted (*p*-value)
Age	0.8	0.02*	0.80	0.014*
Mother education	1.68	0.001***	1.66	<0.001***
Father education	1.58	0.001***	0.53	0.186
Family income	1.95	0.001***	1.04	0.002**
Atopy mother	1.63	0.001***	1.01	<0.001***
Atopy father	1.04	0.002***	0.50	0.281
DNA concentration	1.94	0.001***	1.44	<0.001***
AD status	3.40	0.001***	1.85	<0.001***

Unadjusted and adjusted models report the proportion of variance explained (R^2^) and corresponding *p*-values for each exposure. Adjusted models include all listed exposures simultaneously. Statistically significant associations (adjusted *p*-value) are marked with * for *p* < 0.05, ** for *p* < 0.01, and *** for *p* < 0.001.

### 3.3 Univariate differential abundance analysis (ANCOMBC2)

**FIGURE 3:**
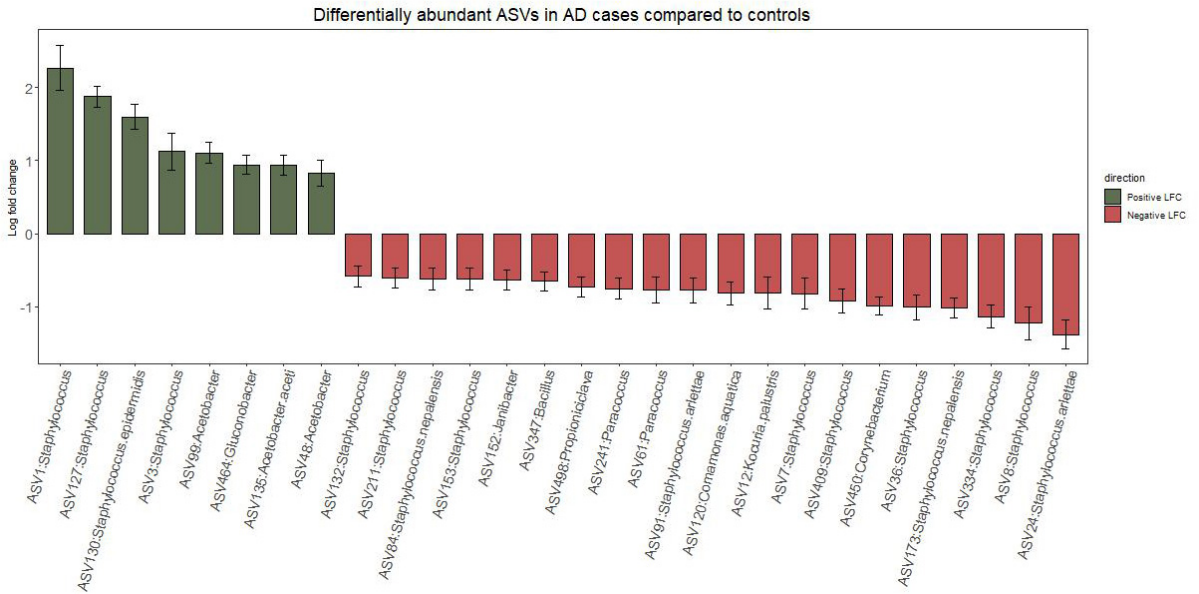
Differentially abundance amplicon sequence variants (ASVs) between lesional atopic dermatitis (AD) skin and controls identified using multivariable ANCOM-BC2. Positive log-fold change values indicate enrichment in AD cases, while negative values represent enrichment in controls. Only ASVs present in ≥20% of samples were included.

**FIGURE 4:**
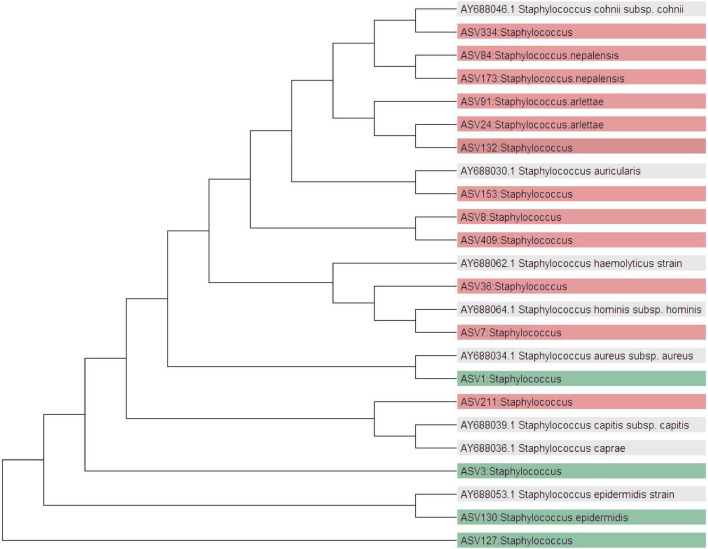
Phylogenetic tree of *Staphylococcus*-assigned amplicon sequence variants (ASVs) and reference strains. ASVs are colored by log-fold change from multivariable ANCOM-BC2 analysis: red indicates enrichment in controls, green in lesional AD skin. Reference sequences (labeled in gray) were included to contextualize ASV placement and infer potential species-level identities.

At genus level, *Acetobacter* and *Staphylococcus* were significantly more abundant in AD cases than in controls (LFC 1.13 and 0.55, respectively; *p* < 0.001). At ASV level, four ASVs belonging to the *Staphylococcus* genus including ASV1 (likely *S. aureus*), ASV127 (likely *S. epidermidis/S. capitis*), ASV130 (*S. epidermidis*), and ASV3(likely *S. epidermidis*) were more abundant in the AD cases (Figure 3). Of these, only ASV130 was called at species level by the DADA 2. In contrast, 12 different ASVs belonging to *Staphylococcus* were found to be more differentially abundant in controls. Four of those were identified down to the species level: ASV91 and ASV24 to *S. arlettae*; ASV84 and ASV173 to *S. nepalensis*. Phylogenetic characterization of *Staphylococcus* assigned ASVs revealed distict species level groupings in lesional AD and controls (Supplementary Table 1 and Figure 4).

### 3.4 Sensitivity analysis

#### 3.4.1 Microbiota profiles in AD children with Toilet seat dermatitis (TSD)

In our dataset, we found that 26% of AD cases presented with TSD. To understand if there were any differences in the microbiota composition in this group, we compared AD cases with and without TSD. TSD was negatively associated with alpha diversity in both uni- and multivariable models. Shannon diversity was significantly reduced in multivariable analysis (β = −0.65, SE = 0.22, adjusted *p* = 0.019), while the negative association with Chao1 richness did not reach statistical significance (β = −12.50, SE = 5.28, adjusted *p* = 0.065) (Supplementary Figure 8 and Supplementary Table 2). At the compositional level, this translated into a slightly higher relative abundance of *Staphylococcus*, accompanied by lower relative abundances of *Paracoccus*, *Roseomonas*, and *Streptococcus* (Supplementary Figure 9).

### 3.4.2 Microbiota profiles in lesional and non-lesional in AD cases

Lesional skin was negatively associated with alpha diversity in both uni- and multivariable models. Shannon diversity was significantly reduced in multivariable analysis (β = 0.52, SE = 0.11, adjusted *p* < 0.001), as was Chao1 richness (β = 13.02, SE = 3.20, adjusted *p* < 0.001) (Supplementary Figure 10 and Supplementary Table 3). At the compositional level, lesional skin showed a higher relative abundance of *Staphylococcus*, while non-lesional skin had more *Paracoccus*, *Roseomonas*, *Kocuria*, *Cutibacterium*, and *Streptococcus* (Supplementary Figure 11).

#### 3.4.3 Microbiota differences and AD severity

Atopic dermatitis severity was negatively associated with alpha diversity in both univariable and multivariable models. In multivariable analysis (mild severity as the reference), Chao1 richness was lower in severe disease (β = −26.50, SE 8.12, adjusted *P* = 0.01) and not significantly lower in moderate disease (β = −10.68, SE 5.44, adjusted *P* = 0.11). Similarly, Shannon diversity was lower in severe disease (β = −0.88, SE 0.29, adjusted *P* = 0.010) and not significantly lower in moderate disease (β = −0.40, SE 0.20, adjusted *P* = 0.10). Diversity was lowest in the severe category after adjustment. (Supplementary Figure 12 and Supplementary Table 4).

## 4 Discussion

In this study we found a lower alpha diversity in AD patients when compared with controls, which was supported by the PERMANOVA analysis. In AD patients, only *S. aureus* and *S. epidermidis* were differentially abundant compared to controls, while the controls harbored a broader range of *Staphylococcus* species. Moreover, we observed a greater non-Staphylococcus diversity in controls, including genera such as *Kocuria*, *Cutibacterium*, *Streptococcus*, and *Roseomonas*. Less commonly reported taxa like *Acetobacter* and *Gluconobacter* were enriched in lesional AD skin. In the univariable analysis (ANCOMB2), we found that several ASVs belonging to the *Staphylococcus* genus had a differential relative abundance in AD cases when compared with controls.

Reduced alpha diversity is a well-documented feature of AD (42). Our results are consistent with previous studies in Western cohorts, where reduced diversity has been associated with greater disease severity and increased susceptibility to *Staphylococcus* colonization (4). However, some reports, particularly in mild or early-stage AD, or in non-lesional skin, have found no significant reduction in diversity, suggesting that diversity may depend on disease activity or sample site (43, 44). Limited studies have assessed alpha diversity in AD while adjusting for a broad range of host, sociodemographic, and technical covariates (42). In our study, alpha diversity remained significantly reduced in lesional skin even after adjustment. We also found an independent association between increasing age and lower diversity, that may suggest bacterial microbiota shifts during childhood maturation (45, 46).

Multivariable PERMANOVA showed that being an AD case contributes to the variation of microbiota composition, in addition to family income, maternal atopy, and DNA concentration. The association with maternal atopy suggests that familial allergic history may shape early microbial colonization through genetic factors (e.g., *FLG* - related barrier alterations that influence the skin microbiota) and early-life vertical transmission (skin-to-skin contact and breast milk-mediated exposures) (42, 47). In addition to genetic factors, studies of cohabitating family members have shown that shared household environments also shape skin microbiota composition, and that both cohabitation and genetic relatedness (e.g., parent-child, siblings) independently contribute to skin microbiota composition (48, 49). These findings suggest that in Indonesia atopic mothers and mother education may influence their child’s skin microbiota not only through genetic susceptibility, but also through shared environmental exposures such as close contact, hygiene routines, and household microbial transmission. The association with DNA concentration is likely due to both technical and biological factors. Higher DNA concentrations often correlate with increased microbial biomass, improving the reliability of diversity measurements in low-biomass samples. Additionally, greater DNA concentrations could reflect an abundance of dominant taxa, such as *Staphylococcus*, which can impact community structure (50).

Differential abundance analysis and consequent phylogenetic analysis showed higher abundance of *Staphylococcus* ASVs clustering closely to *S. epidermidis* and *S. aureus* in cases. These ASVs are well-documented drivers of skin inflammation and barrier disruption in pediatric AD populations (51). In contrast, ASVs most closely clustered with coagulase-negative staphylococci (CoNS) such as *S. cohnii*, *S. saprophyticus*, *S. arlettae*, and *S. lloydii* were more abundant in controls. This included known commensals such as *S. cohnii* and *S. saprophyticus*, which are generally associated with healthy skin (52, 53). While this pattern is not unique to our dataset, it reinforces established observations from European studies that associates these CoNS species with skin health and reduced AD severity (54). Some of these control-associated species may also have clinical relevance. *S. cohnii*, for instance, has demonstrated anti-inflammatory properties in experimental models and has been proposed as a candidate for probiotic-based therapy (52). Other species like *S. arlettae* and *S. lloydii* have been primarily isolated from healthy hosts and are not considered major pathogens, though they may act as opportunists in rare clinical settings (55–57). These findings emphasize that not all *Staphylococcus* species contribute equally to disease and reinforce the value of strain-level resolution in distinguishing pathogenic from commensal members of the skin microbiota.

In addition, we observed an enrichment of less commonly reported genera such as *Acetobacter* and *Gluconobacter* in lesional AD skin. These acetic acid bacteria are rarely documented in skin microbiota studies but are widely found in fermented foods, plants, and humid environments (58). Although not known to be pathogenic in the skin, the consistent detection of these genera after applying a 20% prevalence filter was interesting. However, *Acetobacter* was highly prevalent in air swabs, suggesting environmental contamination rather than true colonization. In contrast, *Gluconobacter* appeared only sporadically in air swabs and was absent from reagent blanks, making contamination less likely. These findings underscore the importance of ecological context and negative controls when interpreting unexpected microbial signatures, and highlight the value of ASV-level resolution in pediatric skin microbiota research.

In the sensitivity analysis, we examined AD participants with clinical features of TSD, a form of localized dermatitis affecting the buttocks and posterior thighs. TSD was prevalent in our study with >20% of AD presenting with this dermatitis, which is most likely associated with hygienic practices involving water spray instead of toilet (41, 59). While overall phylum-level composition appeared visually similar between TSD and non-TSD groups in their lesional sites on forearms and popliteal sites, participants with TSD showed a slight relative increase in *Staphylococcus* abundance and reductions in genera such as *Paracoccus*, *Roseomonas*, and *Streptococcus*. The lower alpha diversity also aligns with a broader AD-associated bacterial imbalance. However, as not all participants with TSD were swabbed at the affected site, namely the gluteal region, we expect these differences to be more pronounced at the affected locations. These findings warrant further investigation as they were purely descriptive and based on a limited subgroup.

## 5 Limitation and strength

A disadvantage of this study is the use of a cross-sectional design, which limits causal inference. Because cross-sectional studies sample participants at a single time point, our design did not include within-patient longitudinal sampling (before, during, and after treatment); therefore, we cannot assess within-individual changes or treatment effects (e.g., potential microbiota restoration with treatment). Additional limitations include the modest sample size. While all controls were swabbed at a consistent anatomical site, the dorsal (extensor) aspect of the distal forearm, lesional samples were taken from different sites depending on disease activity, which may have introduced variability in microbial composition. In addition, all participants were recruited from a single urban tertiary-care referral center to ensure comparable healthcare access and environmental exposures. This recruitment resulted in a cohort largely from high or very high income, more educated households (predominantly urban, with some rural referrals). The narrowed socioeconomic range limits our ability to evaluate socioeconomic effects and reduces generalizability beyond similar settings (e.g., primary/secondary care or rural populations). We also relied on 16S rRNA sequencing, a widely used but limited approach that restricts species-level resolution and functional interpretation. Still, the additional phylogenetic analyses helped us to identify several ASVs up to the species level. Although rigorous quality control measures were applied to ensure robust results, contamination cannot be entirely ruled out, particularly in low-biomass skin swab samples. Environmental genera such as *Acetobacter* and *Gluconobacter* may persist due to external contamination during sample transport. While environmental exposure likely contributes, their biological relevance requires further investigation.

Strengths of this study include ASV-level resolution evaluation and adjustment for a wide range of clinical, sociodemographic, and technical covariates in all analyses. Moreover, studying an underrepresented patient group with an additional analysis of an underrecognized AD presentation TSD, enabled us to extend the current understanding of AD-associated dysbiosis in a tropical pediatric setting and highlight the importance of including diverse populations in microbiota research.

## 6 Conclusion

To conclude, this study confirms key features of AD-associated skin microbiota observed in both European and Asian populations, particularly the dominance of *Staphylococcus aureus* but also *Staphylococcus epidermidis* lesional skin. This pattern is well-documented across global cohorts and was not unique to our study. ASVs analysis showed richer diversity in S species in controls (e.g., *Staphylococcus arlettae*, *Staphylococcus edaphicus*, *Staphylococcus nepalensis*), which are less frequently reported in prior pediatric AD studies from Europe.

## Code availability

The R scripts used for the analysis are available on GitHub at http://github.com/RendyEffendi/SkinMicrobiota.git.

## Data Availability

In accordance with current Indonesian Ministry of Health regulations on the transfer and use of clinical and biological materials, and their associated information and data, the datasets generated and analyzed in this study cannot be deposited in an open international repository without prior approval and a formal Material Transfer and/or Data Sharing Agreement. Anonymized data can be made available for research purposes upon reasonable request to the corresponding author and will be shared following completion of the required approval and agreement process with our institution and the Ministry of Health.
