# Supplementary material for: Profiling skin microbiota in an underrepresented population: Indonesian children with atopic dermatitis and controls
Source: Front Med (Lausanne). 2026 Feb 12;13:1697420. doi: 10.3389/fmed.2026.1697420 (PMC12935604; doi:10.3389/fmed.2026.1697420)
Supplement: Supplementary file 1 [file Table_1.docx]

Supplementary Material

**Figure S1:** Histogram of Decontam Prevalence Threshold **P2**

**Figure S2:** Rarefaction Curve of Sequencing Depth **P3**

**Figure S3:** Flow Diagram Filtering **P4**

**Figure S4:** Relative Abundance at Phylum and Genus Level by Swab Site in AD Cases **P5**

and Controls

**Figure S5:** Relative Abundance of *Staphylococcus* by Sample Type, AD severity, and **P6**

swab sites

**Figure S6**: Core microbiota heatmaps in controls and cases **P7**

**Figure S7:** Beta diversity in AD cases and controls **P8**

**Figure S8:** Alpha Diversity in AD Lesional Skin With and Without Toilet Seat Dermatitis **P9**

**Figure S9:** Genus-Level Microbiota Composition in AD Cases With and **P10**

Without Toilet Seat Dermatitis

**Figure S10:** Alpha Diversity in AD Lesional Skin and Non-lesional **P11**

**Figure S11:** Genus-Level Microbiota Composition in Lesional and Non-Lesional **P12**

Skin Among AD

**Figure S12:** Alpha Diversity Across AD Severity **P13**

**Table S1:** BLAST Results of Key ASVs for Species-Level Identification **P14**

**Table S2:** Multivariable Linear Regression of Alpha Diversity in AD Cases **P17**

by Toilet Seat Dermatitis Status

**Table S3:** Multivariable Linear Regression of Alpha Diversity Among **P18**

Lesional and Non-Lesional Skin in AD Cases

**Table S4:** Multivariable Linear Regression of Alpha Diversity **P19**

Across AD Severity

**Figure S1.** Histogram of Decontam Prevalence Threshold


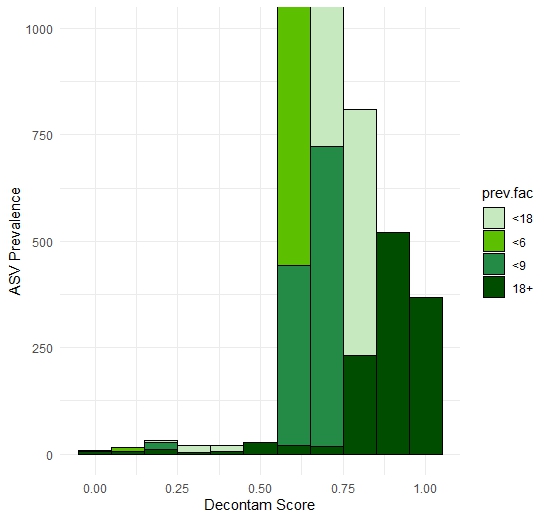


Histogram of ASV Decontam scores calculated using the *prevalence* method from the *Decontam* R package. Bars are colored by prev.fac, a grouping variable based on the number of samples in which each ASV was detected: <6, <9, <18, and 18+. These brackets were empirically derived from the ASV prevalence distribution (1st quartile = 6, median = 9, 3rd quartile = 18) to aid visualization of how detection frequency relates to contaminant classification. A threshold of 0.45 was selected to identify potential contaminants, as it best separated the bimodal score distribution between low-prevalence (likely contaminant) and high-prevalence ASVs.

**Figure S2.** Rarefaction Curve of Sequencing Depth


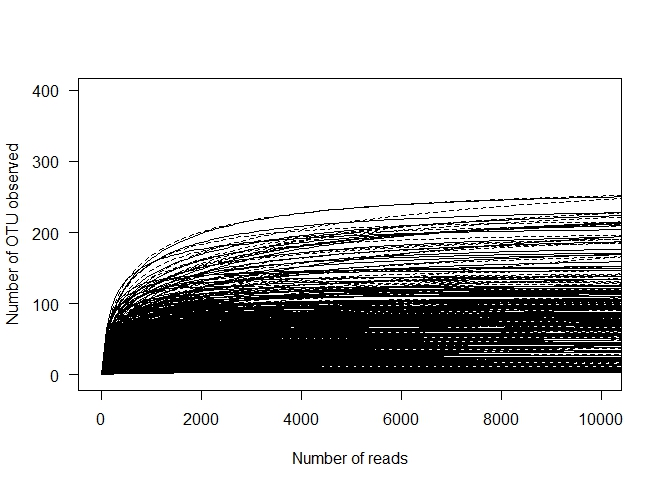
 Rarefaction curves showing the number of observed ASVs as a function of sequencing depth across all samples. Most curves begin to plateau around 2,000 reads, indicating adequate sequencing depth for downstream analysis

.

**Figure S3.** Flow Diagram Filtering


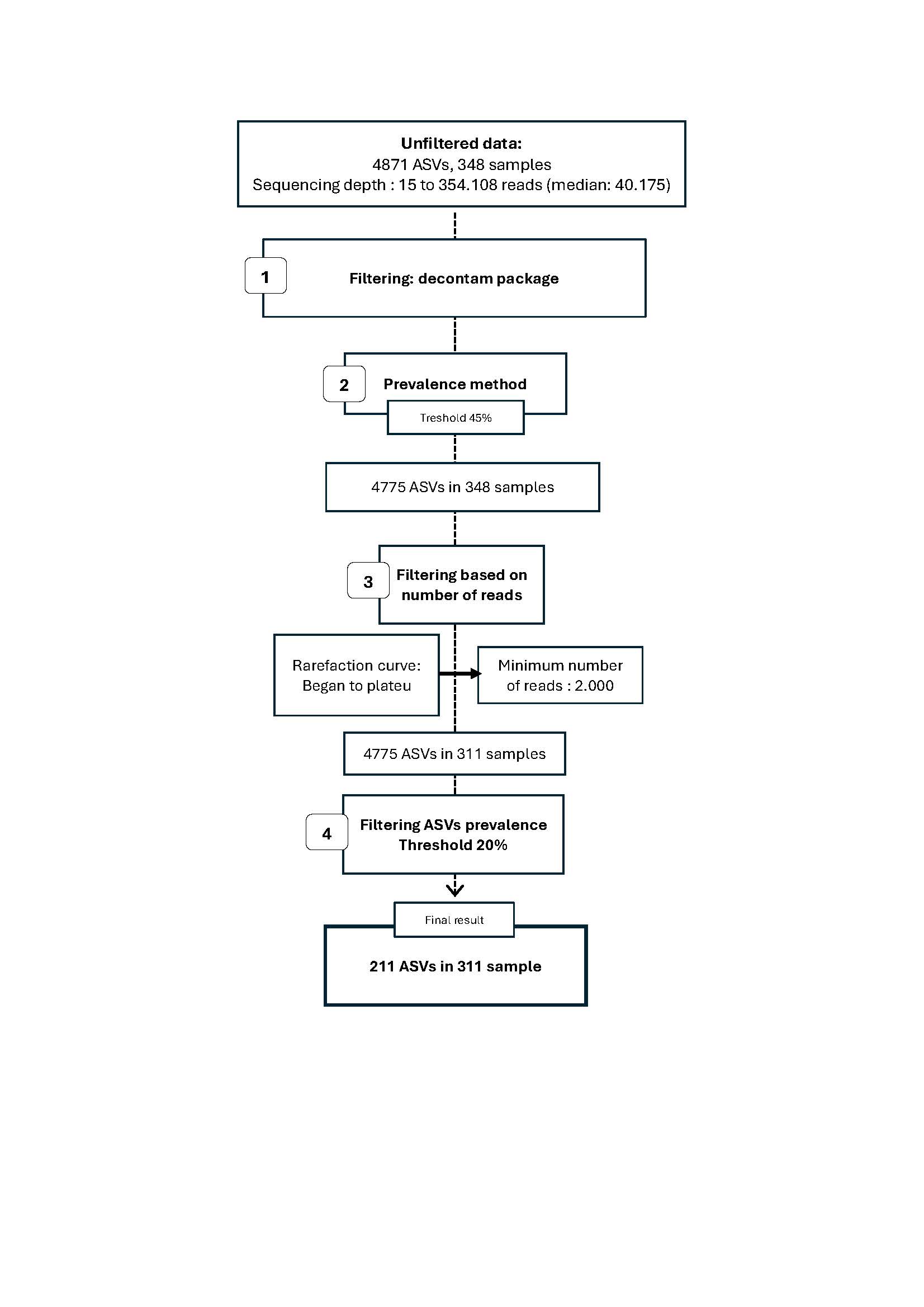


Flow diagram summarizing the four main filtering steps applied to the microbiota dataset. ASVs were first filtered by prevalence from *decontam package*, followed by removal of samples with fewer than 2,000 reads, based on rarefaction curve inspection. A 20% ASV prevalence threshold was then applied. The final dataset included 211 ASVs across 311 samples for downstream analysis.

**Figure S4:** Relative Abundance at Phylum and Genus Level by Swab Site İn AD Cases and Controls


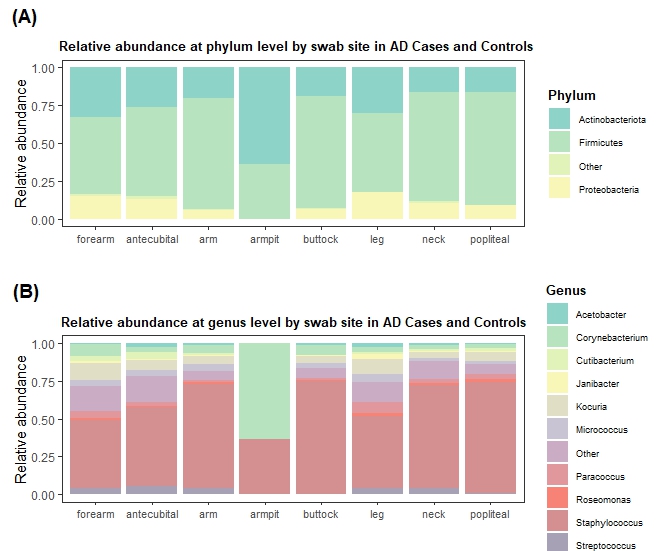


(A) Bar plot showing microbial composition at the phylum level across different swab sites (forearm, antecubital, arm, armpit, buttock, leg, neck, and popliteal). *Firmicutes* and *Actinobacteriota* were dominant across most sites, with *Firmicutes* particularly enriched in the neck, leg, and popliteal regions. (B) Genus-level composition reveals clear dominance of *Staphylococcus* across nearly all swab sites, especially at the popliteal, neck, and leg regions. Other genera such as *Cutibacterium*, *Corynebacterium*, *Roseomonas*, and *Micrococcus* varied in abundance by anatomical site.

**Figure S5.** Relative Abundance of *Staphylococcus* by Sample Type, AD severity, and Swab Sites


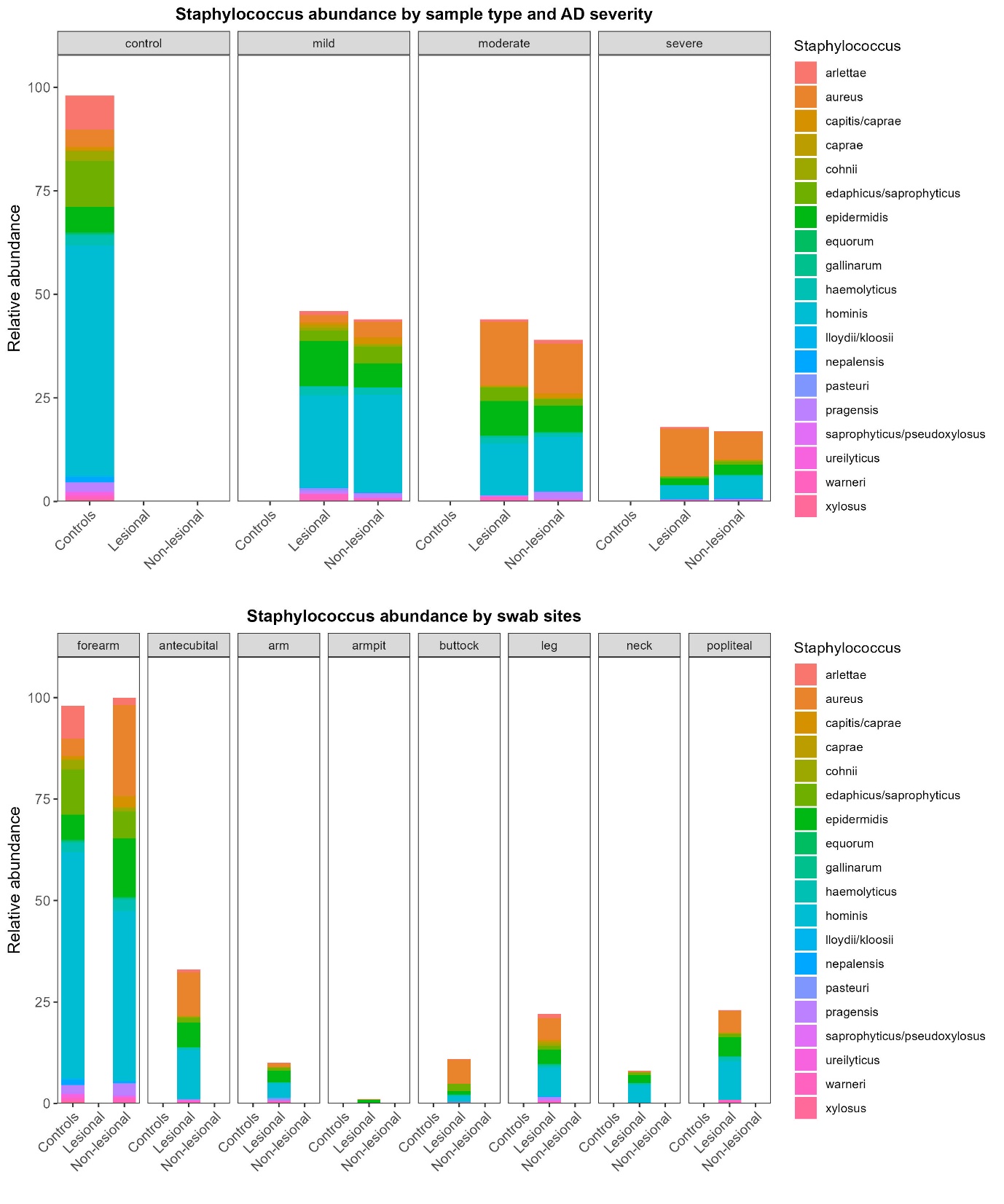


The upper plot displays the relative abundance of *Staphylococcus* across sample types (control, lesional, and non-lesional) and levels of AD severity (control, mild, moderate, and severe). The second plot shows the relative abundance of *Staphylococcus* across various body sites (forearm, antecubital, arm, armpit, buttock, leg, neck, and popliteal). Each bar represents the relative abundance of different Staphylococcus species, with each species color-coded as indicated in the legend.

.

**Figure S6.** Core microbiota heatmaps in controls and cases


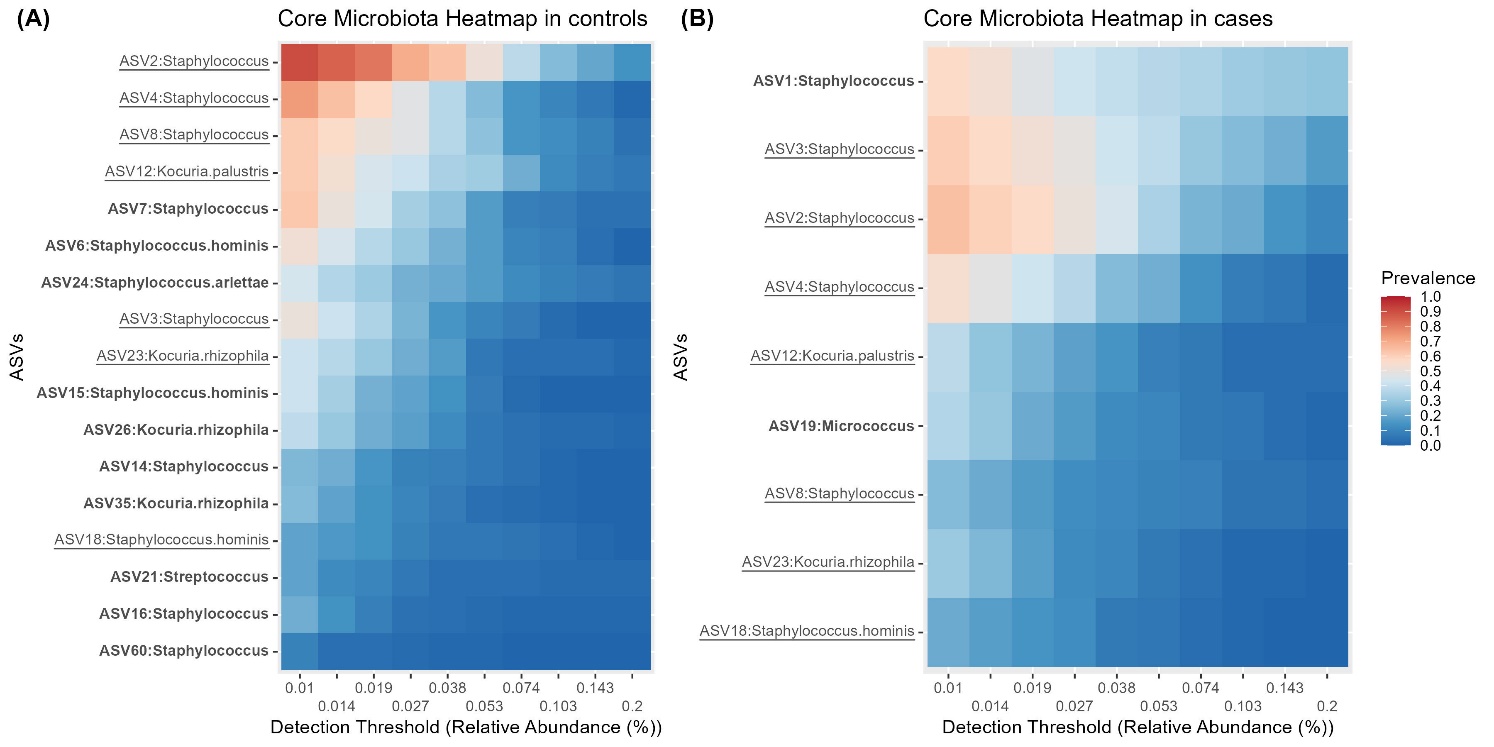


Detection thresholds represent increasing minimum relative abundance values, and cell shading indicates the proportion of samples in which each ASVs was detected. Controls included a greater number of core ASVs, including several *Staphylococcus* and non-*Staphylococcus* ASVs. In contrast, cases was primarily composed of fewer *Staphylococcus* ASVs. Underlined ASVs indicate those shared between both groups, while bolded ASVs are unique to each respective group.

**Figure S7:** Beta diversity in AD cases and controls


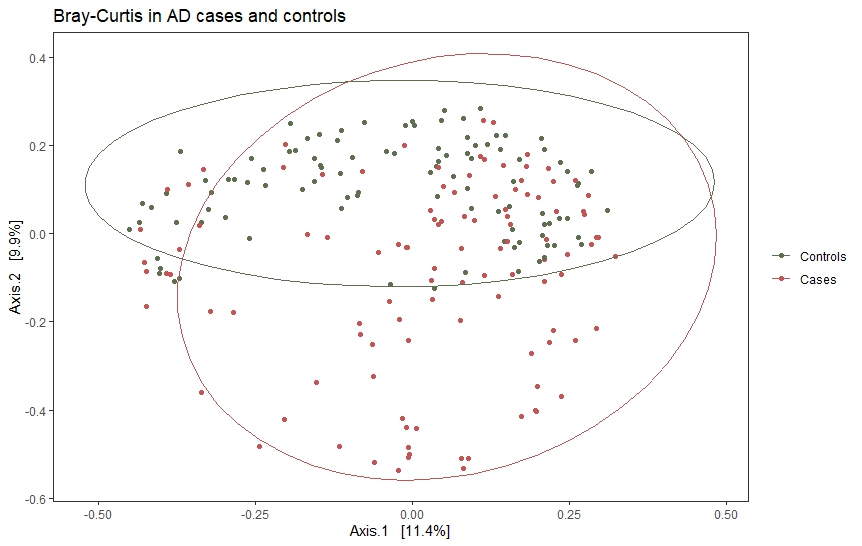


Beta diversity in AD cases and controls**.** Bray–Curtis dissimilarities show clearer separation between groups, with greater dispersion among AD cases (Axis 1 = 11.4%, Axis 2 = 9.9%).

**Figure S8:** Alpha Diversity in AD Lesional Skin With and Without Toilet Seat Dermatitis


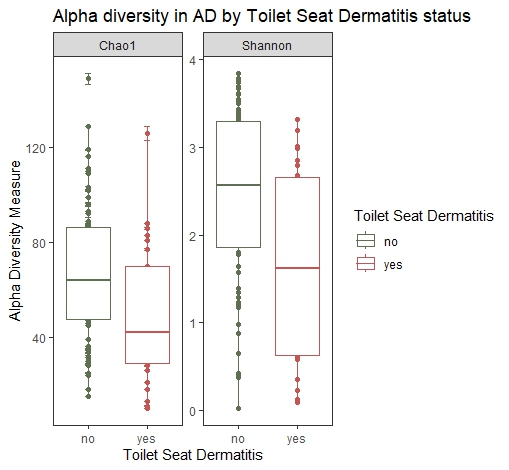


Alpha diversity (Chao1 richness and Shannon diversity) between AD lesional samples with and without TSD. In univariable models, both Chao1 and Shannon indices were significantly lower in the TSD group. In multivariable analysis, Shannon diversity remained significantly reduced, while Chao1 showed a non-significant decreasing trend.

**Figure S9.** Genus-Level Microbiota Composition in AD Cases With and Without Toilet Seat Dermatitis


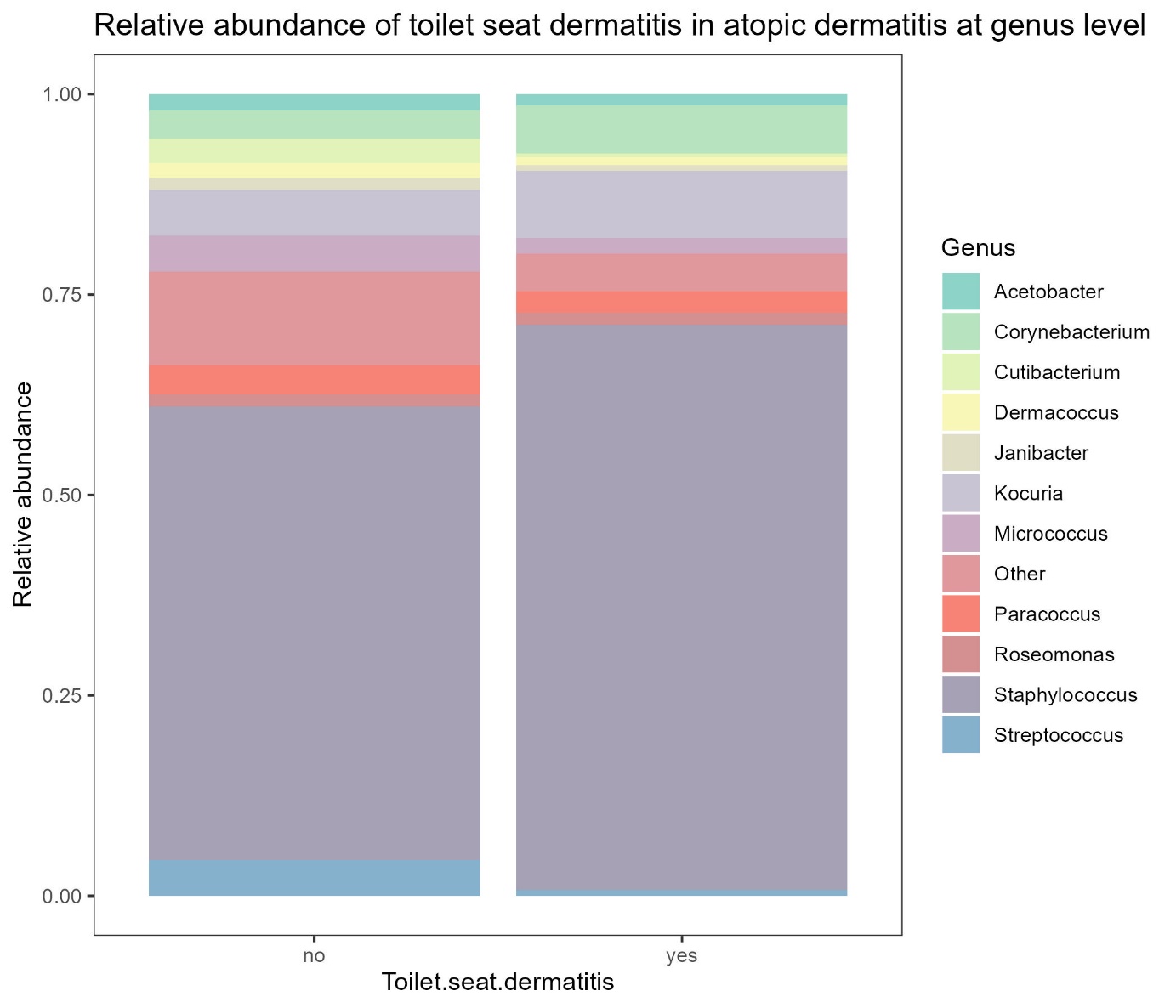


Bar plot showing the relative abundance of bacterial genera among atopic dermatitis (AD) lesional samples stratified by the presence or absence of toilet seat dermatitis (TSD). Samples from participants with TSD showed a higher relative abundance of *Staphylococcus* and lower abundances of *Paracoccus*, *Roseomonas*, and *Streptococcus* compared to those without TSD.

**Figure S10:** Alpha Diversity in AD Lesional Skin and Non-lesional


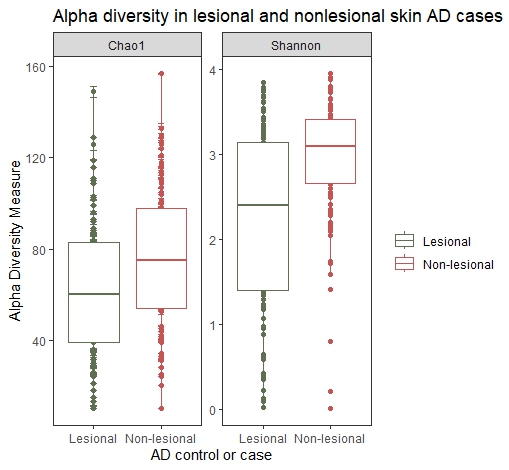


Alpha diversity (Chao1 richness and Shannon diversity) in lesional and non-lesional skin among children with atopic dermatitis (AD). In univariable models, both indices were significantly lower in lesional samples. In multivariable analysis adjusting for age and skin type, both Shannon and Chao1 diversity remained significantly reduced in lesional skin.

**Figure S11:** Genus-Level Microbiota Composition in Lesional and Non-Lesional Skin Among AD


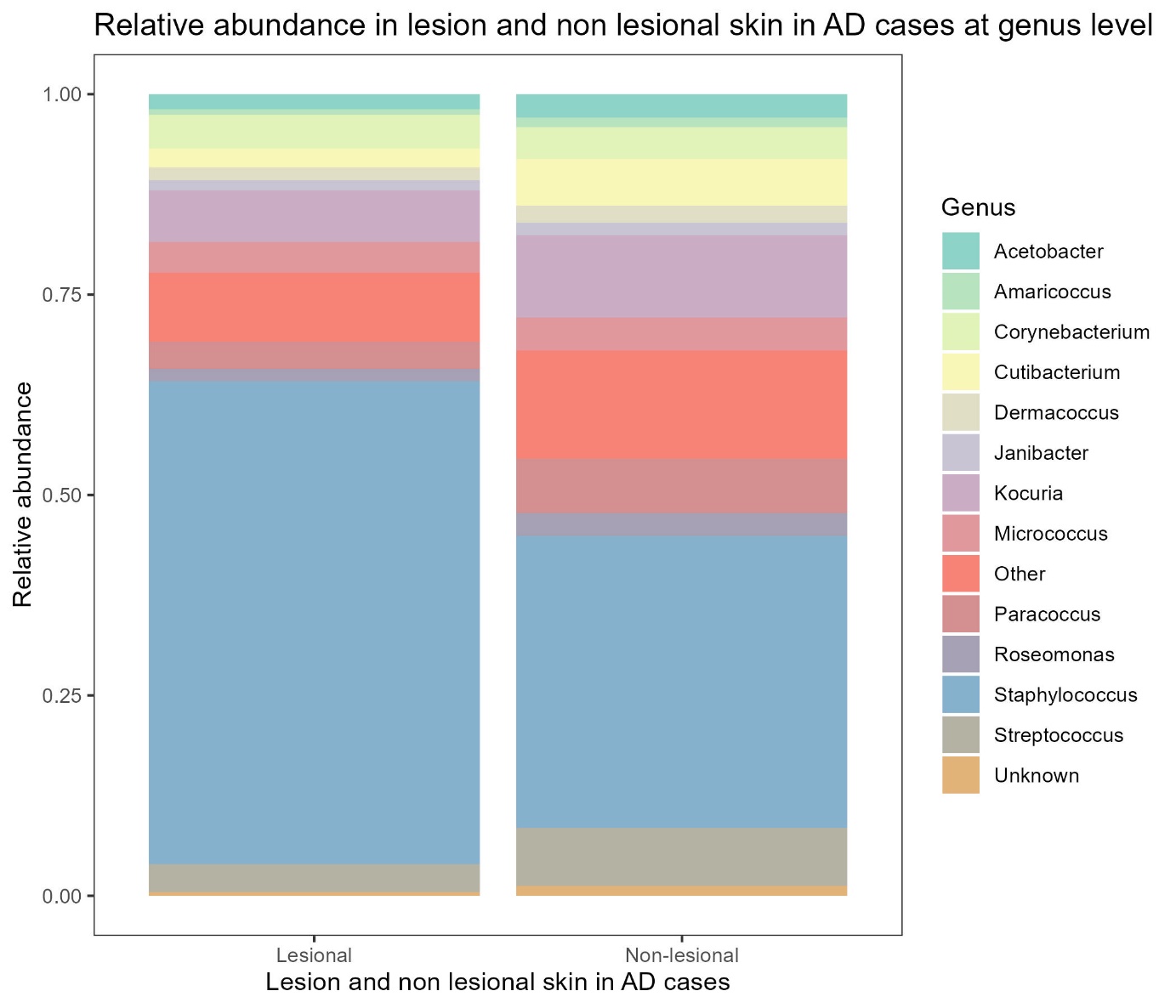


Bar plot showing the relative abundance of bacterial genera in lesional and non-lesional skin samples from children with atopic dermatitis (AD). Lesional samples showed a higher relative abundance of Staphylococcus, whereas non-lesional samples exhibited greater abundances of Paracoccus, Roseomonas, Kocuria, Cutibacterium, and Streptococcus.

**Figure S12:** Alpha Diversity Across AD Severity


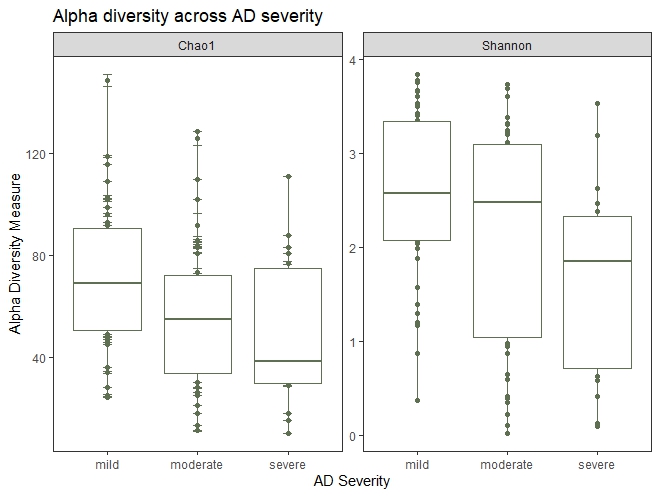


**Table S1.** BLAST Results of Key ASVs for Species-Level Identification

| ASV | Description | Percentage Identical |
| --- | --- | --- |
| ASV1:Staphylococcus | Staphylococcus aureus strain S33 R 16S ribosomal RNA, complete sequence | 100% |
|  | Staphylococcus aureus strain NBRC 100910 16S ribosomal RNA gene, partial sequence | 100% |
|  | Staphylococcus aureus strain MVF-7 16S ribosomal RNA, partial sequence | 100% |
|  | Staphylococcus aureus strain ATCC 12600 16S ribosomal RNA, partial sequence | 100% |
| ASV2: Staphylococcus | Staphylococcus hominis strain DM 122 16S ribosomal RNA, partial sequence | 99.80% |
| ASV3:Staphylococcus | Staphylococcus epidermidis strain NBRC 100911 16S ribosomal RNA, partial sequence | 100% |
|  | Staphylococcus epidermidis strain Fussel 16S ribosomal RNA, partial sequence | 100% |
| ASV4: Staphylococcus | Staphylococcus hominis strain DM 122 16S ribosomal RNA, partial sequence | 100% |
| ASV7:Staphylococcus | Staphylococcus hominis strain DM 122 16S ribosomal RNA, partial sequence | 99.80% |
|  | Staphylococcus hominis subsp. novobiosepticus strain GTC 1228 16S ribosomal RNA, partial sequence | 99.19% |
| ASV8:Staphylococcus | Staphylococcus edaphicus strain CCM 8730 16S ribosomal RNA, partial sequence | 100% |
|  | Staphylococcus saprophyticus subsp. saprophyticus ATCC 15305 = NCTC 7292 16S ribosomal RNA, partial sequence | 100% |
| ASV12:Kocuria.palustris | Kocuria palustris strain TAGA27 16S ribosomal RNA, partial sequence | 100% |
| ASV18: Staphylococcus | Staphylococcus hominis strain DM 122 16S ribosomal RNA, partial sequence | 99.60% |
| ASV24:Staphylococcus.arlettae | Staphylococcus.arlettae strain ATCC 43957 16S ribosomal RNA, partial sequence | 100% |
| ASV36:Staphylococcus | Staphylococcus hominis strain DM 122 16S ribosomal RNA, partial sequence | 99.60% |
|  | Staphylococcus borealis strain 51-48 16S ribosomal RNA, partial sequence | 99.00% |
| ASV48:Acetobacter | Acetobacter tropicalis strain NBRC 16470 16S ribosomal RNA, partial sequence | 100% |
| ASV61:Paracoccus | Paracoccus haeundaensis strain BC74171 16S ribosomal RNA, partial sequence | 100% |
|  | Paracoccus carotinifaciens strain E-396 16S ribosomal RNA, partial sequence | 100% |
|  | Paracoccus marcusii strain MH1 16S ribosomal RNA, partial sequence | 100% |
| ASV84:Staphylococcus.nepalensis | Staphylococcus nepalensis strain CW1 16S ribosomal RNA, partial sequence | 100% |
| ASV91:Staphylococcus.arlettae | Staphylococcus.arlettae strain ATCC 43957 16S ribosomal RNA, partial sequence | 99.80% |
| ASV99:Acetobacter | Acetobacter aceti NBRC 14818 16S ribosomal RNA, partial sequence | 99.54% |
|  | Acetobacter aceti NBRC 14818 strain JCM 7641 16S ribosomal RNA, partial sequence | 99.54% |
|  | Acetobacter sicerae strain LMG 1531 16S ribosomal RNA, partial sequence | 99.31% |
| ASV120:Comamonas.aquatica | Comamonas aquatica strain LMG 2370 16S ribosomal RNA, partial sequence | 100% |
|  | Comamonas aquatica subsp. rana strain CW-25 16S ribosomal RNA, partial sequence | 100% |
| ASV127:Staphylococcus | Staphylococcus epidermidis strain NBRC 100911 16S ribosomal RNA, partial sequence | 99.60% |
|  | Staphylococcus epidermidis strain Fussel 16S ribosomal RNA, partial sequence | 99.60% |
|  | Staphylococcus capitis strain JCM 2420 16S ribosomal RNA, partial sequence | 98.59% |
|  | Staphylococcus capitis subsp. urealyticus strain MAW 8436 16S ribosomal RNA, partial sequence | 98.59% |
| ASV130:Staphylococcus.epidermidis | Staphylococcus epidermidis strain NBRC 100911 16S ribosomal RNA, partial sequence | 99.80% |
|  | Staphylococcus epidermidis strain Fussel 16S ribosomal RNA, partial sequence | 99.80% |
| ASV132:Staphylococcus | Staphylococcus arlettae strain ATCC 43957 16S ribosomal RNA, partial sequence | 99.80% |
|  | Staphylococcus ureilyticus strain CK27 16S ribosomal RNA, partial sequence | 98.80% |
|  | Staphylococcus gallinarum strain VIII1 16S ribosomal RNA, partial sequence | 98.59% |
| ASV135:Acetobacter.aceti | Acetobacter aceti NBRC 14818 16S ribosomal RNA, partial sequence | 100% |
|  | Acetobacter aceti NBRC 14818 strain JCM 7641 16S ribosomal RNA, partial sequence | 100% |
| ASV152:Janibacter | Janibacter alkaliphilus strain SCSIO 10480 16S ribosomal RNA, partial sequence | 99.55% |
| ASV153:Staphylococcus | Staphylococcus lloydii strain 23_2_7_LY 16S ribosomal RNA, partial sequence | 100% |
|  | Staphylococcus kloosii strain ATCC 43959 16S ribosomal RNA, partial sequence | 100% |
| ASV173:Staphylococcus.nepalensis | Staphylococcus nepalensis strain CW1 16S ribosomal RNA, partial sequence | 99.79% |
| ASV211:Staphylococcus | Staphylococcus capitis strain JCM 2420 16S ribosomal RNA, partial sequence | 99.80% |
|  | Staphylococcus caprae strain ATCC 35538 16S ribosomal RNA, partial sequence | 99.80% |
| ASV241:Paracoccus | Paracoccus spongiarum strain 2205BS29-5 16S ribosomal RNA, partial sequence | 99.77% |
|  | Paracoccus amoyensis strain 11-3 16S ribosomal RNA, partial sequence | 99.77% |
|  | Paracoccus caeni strain MJ17 16S ribosomal RNA, partial sequence | 99.54% |
| ASV334:Staphylococcus | Staphylococcus cohnii strain GH 137 16S ribosomal RNA, partial sequence | 100% |
| ASV347:Bacillus | Bacillus paramycoides strain MCCC 1A04098 16S ribosomal RNA, partial sequence | 100% |
|  | Bacillus albus strain MCCC 1A02146 16S ribosomal RNA, partial sequence | 100% |
|  | Bacillus cereus strain JCM 2152 16S ribosomal RNA, partial sequence | 100% |
|  | Bacillus cereus strain CCM 2010 16S ribosomal RNA, partial sequence | 100% |
|  | Bacillus cereus ATCC 14579 16S ribosomal RNA (rrnA), partial sequence | 100% |
|  | Bacillus cereus strain IAM 12605 16S ribosomal RNA, partial sequence | 100% |
|  | Bacillus cereus ATCC 14579 16S ribosomal RNA, partial sequence | 100% |
|  | Bacillus cereus strain NBRC 15305 16S ribosomal RNA, partial sequence | 100% |
| ASV409:Staphylococcus | Staphylococcus edaphicus strain CCM 8730 16S ribosomal RNA, partial sequence | 99.80% |
|  | Staphylococcus saprophyticus subsp. saprophyticus ATCC 15305 = NCTC 7292 16S ribosomal RNA, partial sequence | 99.80% |
| ASV450:Corynebacterium | Corynebacterium variabile strain DSM 20132 16S ribosomal RNA, partial sequence | 98.08% |
|  | Corynebacterium neomassiliense strain Marseille-P3888 16S ribosomal RNA, partial sequence | 97.65% |
|  | Corynebacterium provencense strain SN15 16S ribosomal RNA, partial sequence | 97.65% |
| ASV464:Gluconobacter | Gluconobacter roseus NBRC 3990 16S ribosomal RNA, partial sequence | 100% |
|  | Gluconobacter vitians strain LMG 31484 16S ribosomal RNA, partial sequence | 100% |
|  | Gluconobacter oxydans strain DSM 3503 16S ribosomal RNA, partial sequence | 100% |
| ASV498:Propioniciclava | Propioniciclava sinopodophylli strain TEYR-7 16S ribosomal RNA, partial sequence | 95.92% |
|  | Propioniciclava flava strain VG341 16S ribosomal RNA, partial sequence | 92.93% |
|  | Propioniciclava coleopterorum strain HDW11 16S ribosomal RNA, partial sequence | 91.63% |

BLAST searches were conducted on 23 June 2025 to identify the closest species-level matches for selected amplicon sequence variants (ASVs) based on 16S rRNA gene sequences. The table lists the top matches with the highest percentage identity per ASV. Only matches with 100% identity or the highest available match (if <100%) are reported.

**Table S2.** Multivariable Linear Regression of Alpha Diversity in AD Cases by Toilet Seat Dermatitis Status

| **Tested exposure** | **Chao1 richness** | | | | | | | **Shannon diversity** | | | | |
| --- | --- | --- | --- | --- | --- | --- | --- | --- | --- | --- | --- | --- |
|  | | | **β** | **SE** | | **Adjusted p** | | **β** | | **SE** | **Adjusted p** | |
| Toilet seat dermatitis (ref:no) | | -12.50 | | | 5.28 | | 0.065 | | -0.65 | 0.22 | | 0.019* |
| Age in years | | -1.45 | | | 0.72 | | 0.103 | | -0.07 | 0.03 | | 0.079 |
| Father education high (ref:intermediate) | | 0.74 | | | 6.84 | | 0.926 | | 0.003 | 0.28 | | 0.991 |
| Mother education high (ref:intermediate) | | -2.89 | | | 6.49 | | 0.854 | | -0.19 | 0.27 | | 0.699 |
| Family income very high (ref:high) | | -14.21 | | | 4.98 | | 0.02* | | -0.37 | 0.20 | | 0.158 |
| Birth method section caesarea (ref: vaginal) | | -5.22 | | | 4.76 | | 0.511 | | -0.16 | 0.19 | | 0.699 |
| Exclusive breastfeed no (ref: yes) | | 0.67 | | | 4.66 | | 0.926 | | 0.11 | 0.19 | | 0.699 |
| Feeding habit – hand feed (ref:spoon feed) | | -5.50 | | | 5.98 | | 0.585 | | -0.07 | 0.24 | | 0.830 |
| Atopy mother yes (ref:no) | | -9.69 | | | 4.83 | | 0.103 | | -0.12 | 0.20 | | 0.699 |
| Atopy Father yes (ref:no) | | 2.95 | | | 5.11 | | 0.816 | | 0.11 | 0.21 | | 0.699 |
| DNA concentration | | -0.85 | | | 0.20 | | <0.001*** | | -0.02 | 0.01 | | 0.019* |

Multivariable linear regression results assessing associations between toilet seat dermatitis and alpha diversity (Chao1 richness and Shannon diversity) among children with AD. Beta coefficients (β), standard errors (SE), and adjusted p-values are shown. All models were restricted to AD cases and adjusted for relevant covariates. Statistically significant results (adjusted p-value) are marked with * for p < 0.05, ** for p < 0.01, and *** for p < 0.001.

**Table S3.** Multivariable Linear Regression of Alpha Diversity Among Lesional and

Non-Lesional Skin in AD Cases

| **Tested exposure** | **Chao1 richness** | | | | | | | **Shannon diversity** | | | | | |
| --- | --- | --- | --- | --- | --- | --- | --- | --- | --- | --- | --- | --- | --- |
|  | | | **β** | **SE** | | **Adjusted p** | | **β** | | **SE** | **Adjusted p** | | |
| Non-lesional (ref:lesional) | | 13.02 | | | 3.20 | | <0.001* | | -0.52 | 0.12 | | | <0.001* |
| Age in years | | -1.59 | | | 0.49 | | 0.004** | | -0.07 | 0.02 | | | <0.001* |
| Father education high (ref:intermediate) | | 4.55 | | | 4.92 | | 0.421 | | 0.14 | 0.18 | | | 0.532 |
| Mother education high (ref:intermediate) | | -0.88 | | | 4.58 | | 0.918 | | -0.09 | 0.17 | | | 0.661 |
| Family income very high (ref:high) | | -10.06 | | | 3.63 | | 0.013* | | -0.35 | 0.13 | | | 0.023* |
| Birth method section caesarea (ref: vaginal) | | -6.84 | | | 3.42 | | 0.087 | | -0.14 | 0.13 | | | 0.485 |
| Exclusive breastfeed no (ref: yes) | | 4.37 | | | 3.32 | | 0.274 | | 0.21 | 0.12 | | | 0.198 |
| Feeding habit – hand feed (ref:spoon feed) | | -4.30 | | | 4.20 | | 0.399 | | -0.12 | 0.15 | | | 0.532 |
| Atopy mother yes (ref:no) | | -5.72 | | | 3.46 | | 0.162 | | -0.11 | 0.13 | | | 0.532 |
| Atopy Father yes (ref:no) | | -0.10 | | | 3.56 | | 0.978 | | -0.01 | 0.13 | | | 0.910 |
| DNA concentration | | -1.13 | | | 0.19 | | <0.001*** | | -0.03 | 0.01 | | <0.001*** | |

Multivariable linear regression results assessing associations between lesional and non-lesional skin and alpha diversity (Chao1 richness and Shannon diversity) among children with AD. Beta coefficients (β), standard errors (SE), and adjusted p-values are shown. All models were restricted to AD cases and adjusted for relevant covariates. Statistically significant results (adjusted p-value) are marked with * for p < 0.05, ** for p < 0.01, and *** for p < 0.001.

**Table S4.** Multivariable Linear Regression of Alpha Diversity Across AD Severity

| **Tested exposure** | **Chao1 richness** | | | | | | | **Shannon diversity** | | | | | |
| --- | --- | --- | --- | --- | --- | --- | --- | --- | --- | --- | --- | --- | --- |
|  | | | **β** | **SE** | | **Adjusted p** | | **β** | | **SE** | **Adjusted p** | | |
| Moderate severity (ref:mild) | | -10.68 | | | 5.44 | | 0.11 | | -0.40 | 0.20 | | | 0.10 |
| Severe severity (ref:mild) | | -26.50 | | | 8.12 | | 0.01** | | -0.88 | 0.29 | | | 0.016** |
| Age in years | | -0.74 | | | 0.81 | | 0.426 | | -0.06 | 0.02 | | | 0.074 |
| Father education high (ref:intermediate) | | -7.57 | | | 8.02 | | 0.426 | | -0.21 | 0.29 | | | 0.619 |
| Mother education high (ref:intermediate) | | -6.20 | | | 7.24 | | 0.426 | | -0.07 | 0.26 | | | 0.854 |
| Family income very high (ref:high) | | -15.91 | | | 5.66 | | 0.02* | | -0.41 | 0.20 | | | 0.109 |
| Birth method section caesarea (ref: vaginal) | | -7.90 | | | 5.31 | | 0.260 | | -0.13 | 0.19 | | | 0.619 |
| Exclusive breastfeed no (ref: yes) | | -1.44 | | | 5.38 | | 0.788 | | 0.02 | 0.19 | | | 0.884 |
| Feeding habit – hand feed (ref:spoon feed) | | -6.01 | | | 6.58 | | 0.426 | | -0.17 | 0.24 | | | 0.619 |
| Atopy mother yes (ref:no) | | -10.53 | | | 5.41 | | 0.117 | | -0.23 | 0.19 | | | 0.451 |
| Atopy Father yes (ref:no) | | 6.02 | | | 5.50 | | 0.426 | | -0.06 | 0.20 | | | 0.854 |
| DNA concentration | | -0.56 | | | 0.217 | | 0.03* | | -0.02 | 0.007 | | 0.004*** | |

Multivariable linear regression results assessing associations between AD severity and alpha diversity (Chao1 richness and Shannon diversity) among children with AD. Beta coefficients (β), standard errors (SE), and adjusted p-values are shown. All models were restricted to AD cases and adjusted for relevant covariates. Statistically significant results (adjusted p-value) are marked with * for p < 0.05, ** for p < 0.01, and *** for p < 0.001.
